# Sex-specific ranges and ratios for anogenital distance among Thai full-term newborns 

**DOI:** 10.1186/s12887-022-03325-y

**Published:** 2022-05-10

**Authors:** Nattakarn Numsriskulrat, Khomsak Srilanchakon, Chaiyat Pronprechatham, Sopon Pornkunwilai, Vichit Supornsilchai

**Affiliations:** 1 Division of Pediatric Endocrinology, Department of Pediatrics, Faculty of Medicine, Chulalongkorn University, Bangkok, 10330 Thailand; 2 Police General Hospital, Bangkok, Thailand

**Keywords:** Anogenital distance (AGD) ratio, Anopenile distance (AGD_AP_), Anoscrotal distance (AGD_AS_), Anoclitoral distance (AGD_AC_), Anofourchette distance (AGD_AF_)

## Abstract

**Introduction:**

Anogenital distance (AGD) is a marker of prenatal androgen exposure and a tool for assessment of differences of sex development. Data for AGD in newborns have been published, but these findings may not be applicable to Thai newborns.

**Aim:**

To provide the sex-specific ranges for AGD in Thai full-term newborns.

**Methods:**

A cross-sectional study was conducted in term newborns in Thailand, during 2016–2018. AGD was measured from anus to anterior base of penis (AGD_AP_) and to perineoscrotal junction (AGD_AS_) in males and from anus to clitoris (AGD_AC_) and to posterior fourchette (AGD_AF_) in females. AGD ratio is defined as AGD_AS_ divided by AGD_AP_ in males and AGD_AF_ divided by AGD_AC_ in females.

**Results:**

A total of 364 newborns were studied (male 51.4%). The mean AGD_AS_, AGD_AP_ and AGD ratio in males were 25.20 ± 4.80, 52.60 ± 6.90 and 0.48 ± 0.08 mm, respectively. The mean AGD_AF_, AGD_AC_, and AGD ratio in females were 16.50 ± 3.90, 42.60 ± 6.20 and 0.39 ± 0.08 mm, respectively. There were significant differences between AGD_AS_ and AGD_AF_, AGD_AP_ and AGD_AC_, and AGD ratio between males and females (*p* < 0.001). The AGD_AS_, AGD_AP_, AGD_AF_, AGD_AC_ were correlated with birth weight and length, but AGD ratio showed no correlation.

**Conclusion:**

The sex-specific ranges for AGD in Thai full-term newborns were determined. AGD ratio is a useful marker of prenatal androgen exposure since it differs between sexes, but constant between races and did not vary by body size.

## What is already known on this topic

- 1. Anogenital distance is a marker for prenatal androgen exposure during the critical period of external genital development (8th-16th week of gestation).

## What this paper adds

- 1. The AGD ratio is a more reliable indicator of prenatal androgen exposure than other AGD parameters since it is consistent between races, different between sexes, and unrelated to anthropometrics.
- 2. Reference ranges of AGD in healthy Thai newborn.

## Introduction

The distance between the anus and genitalia is referred to as the anogenital distance (AGD), which serves as a marker of prenatal androgen exposure [1]. Under-androgenization or over-androgenization can be inferred indirectly from AGD. For example, an under-androgenized newborn has a shorter AGD than normal [1]. Male offspring born to polycystic ovary syndrome (PCOS) mothers are more likely to have longer AGD due to increased androgen levels during the 8^th^-16^th^ gestation weeks during pregnancy which is the critical period of external genitalia development [2]. AGD is also longer in androgenized females due to labioscrotal fusion, as demonstrated in neonates with virilized congenital adrenal hyperplasia [3].

Gender, gestational age, and anthropometric characteristics including birth weight and length are all significant correlates to AGD [4, 5]. AGD is becoming more widely used in clinical settings to assess potential reproductive dysfunction. Since AGD can also be affected by endocrine disruptors, it has been utilized in environmental toxicology to assess the health implications of chemicals such as Bisphenol A (BPA) and Polychlorinated Bisphenyls (PCBs) with endocrine-altering capabilities [6]. While data exists on Caucasian and selected Asian newborn populations, there is a paucity of data among newborns in Thailand to determine normal distance ranges and ratios by gender. In this study, the sex-specific reference ranges for AGD in healthy Thai full-term newborns were presented including comparisons to previous studies both locally and worldwide.

## Materials and methods

### Participants

A cross-sectional study was undertaken among newborns in Thailand from 2016 to 2018. Healthy newborns aged zero to 72 h were enrolled in the study. Exclusion factors included the presence of genital ambiguity, dysmorphic features, and known maternal ingestion of androgenic medications or substances.

The recommended minimum sample size to determine statistically significance was calculated using the formula $\mathrm{N}={(\mathrm{z\sigma }/\mathrm{E})}^{2}$ [7].

Z = the value from the standard normal distribution reflecting the confidence level (Z = 1.96 for 95%).

σ = standard deviation from the reference study.

E = desired margin of error.

To estimate the SD for the present study, we used data published from a reference study where the mean anoscrotal distance was 24.7 ± 4.5 mm [8]. A margin of error of 0.5 mm was considered.

$$\mathrm N={(1.96(4.5)/0.5)}^2\;=\;312$$

Anthropometric measurements of birth weight, supine length, head and thoracic circumferences and AGD of all newborns were performed by well-trained physicians. Weight was measured using a digital infant scale with clothing and diapers removed to the nearest 0.01 kg. With a Harpenden Infantometer, length was measured to the nearest 0.1 cm. The newborn was placed naked on the infantometer in a supine position, the head was held against the immovable head-board board by an assistant, the knees were held together and held down against the board surface, and the heels were also held down to ensure the child's body and pelvis were straight along the measuring device, the foot-board was then drawn up to meet the heels and this was measured as the length. A flexible measuring tape was used to obtain head and thoracic circumferences. A complete physical examination of each baby occurred in a warmed environment. The obstetric history of each child was obtained.

### Genital distance measurements

A digital sliding caliper (SuperCaliper SERIES 500, Mitutoyo, Thailand) was used to measure AGD to the nearest 0.1 mm. Newborns were placed in a supine position. An assistant held both hips in flexion, flexed and pulled the knees back towards the shoulders. The caliper was positioned adjacent to the surface of the genitalia, digital screen turned away from the researcher, and the single AGD measurement was obtained.

**Fig. 1:**
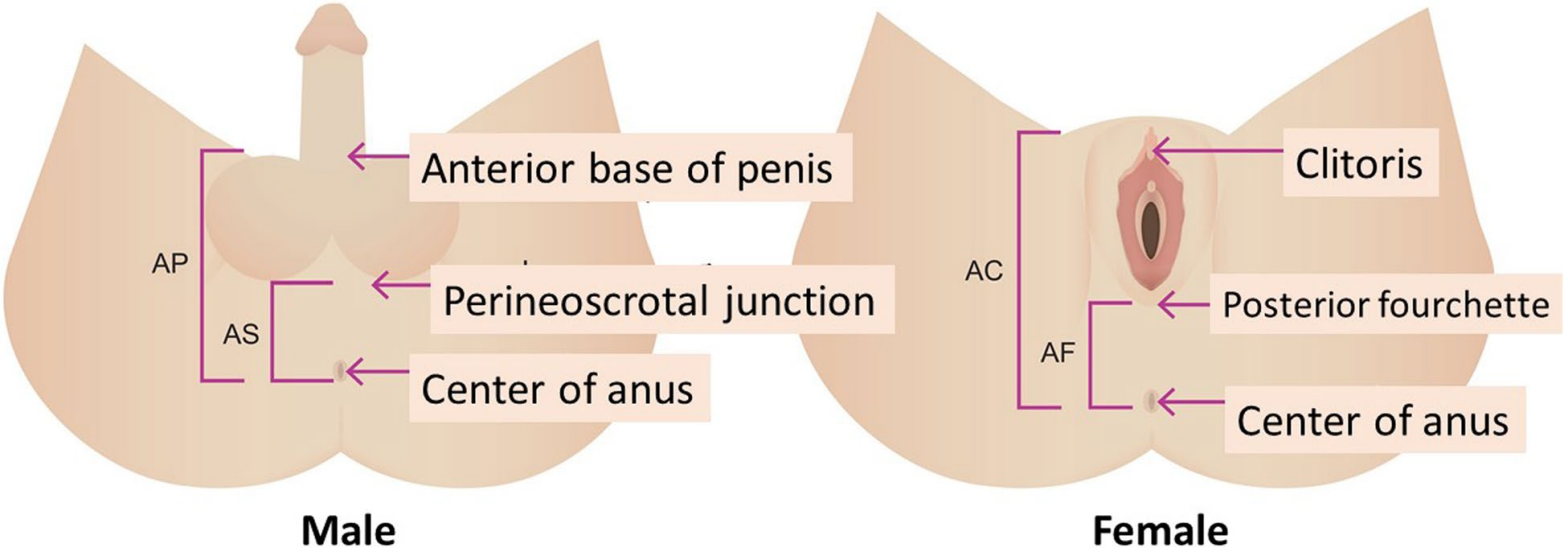
Anogenital distance measurement. AP: distance from anus to anterior base of penis. AS: distance from anus to perineoscrotal junction. AC: distance from anus to clitoris. AF: distance from anus to posterior fourchette

In males, AGD was measured from the center of the anus to the anterior base of the penis (anopenile distance, AGD_AP_) and to the perineoscrotal junction (anoscrotal distance, AGD_AS_). In females, AGD was measured from the center of the anus to the clitoris (anoclitoral distance, AGD_AC_) and to the posterior fourchette (anofourchette distance, AGD_AF_) (Fig. 1). The anogenital distance ratio (AGD ratio) is calculated as AGD_AS_ divided by AGD_AP_ in males and AGD_AF_ divided by AGD_AC_ in females.

### Statistical analysis

For continuous variables, we calculated means and standard deviations, whereas for categorical variables, we calculated frequencies and percentages. The unpaired t-test was used to test for means differences between continuous variables. Pearson’s correlation coefficient (r) assessed the correlation between continuous variables. Associations between variables were evaluated using linear regression. A *p*-value < 0.05 was considered statistically significant. All statistical data were analyzed using SPSS software version 22 (SPSS, Inc., Chicago, IL, USA).

## Results

A total of 364 newborns were included in the study. There were 187 male newborns (51.4%) and 177 female newborns (48.6%). The mean ± SD gestational age was 38.69 ± 1.09 weeks (range, 37–41 weeks). Anthropometrics data including weight, length, and head circumference are not significantly different between males and females by Student’s t-test. The mean ± SD AGD_AS_, AGD_AP_ and AGD ratio in males were 25.20 ± 4.80, 52.60 ± 6.90 and 0.48 ± 0.08 mm, respectively. The mean AGD_AF_, AGD_AC_, and AGD ratio in females were 16.5 ± 3.9, 42.6 ± 6.2 and 0.39 ± 0.08 mm, respectively (Table 1). From the Student’s t-test, there were significant difference between AGD_AS_ and AGD_AF_, AGD_AP_ and AGD_AC_, and AGD ratio between males and females (*p* < 0.001). The AGD percentiles of subjects are shown in Table 2 at five percentiles (3%, 10%, 50%, 90%, 97%).

**Table 1:** Anthropometric parameters and anogenital distances of the participants (*N* = 364)

	**Mean**	**SD**	**Median**	**Min**	**Max**
**Birth weight, gm**					
Male	3190.85	390.28	3150.00	2330.00	4380.00
Female	3151.29	414.30	3110.00	2260.00	4400.00
**Length, cm**					
Male	51.4	1.9	51.0	45.5	57.0
Female	51.0	2.0	51.0	47.0	57.0
**Head circumference, cm**					
Male	33.4	1.3	33.5	23.5	37.0
Female	33.2	1.2	33.0	30.0	37.0
**Chest circumference, cm**					
Male	32.4	1.49.0	32.0	29.0	37.0
Female	32.3	1.52.0	32.0	29.0	37.0
**Anogenital distances**					
**Male (*N* = 187)**					
AGD_AS_*, mm	25.2	4.8	25.0	10.0	40.0
AGD_AP_**, mm	52.6	6.9	50.0	34.0	80.0
AGD ratio***	0.48	0.08	0.50	0.25	0.67
**Female (*N* = 177)**					
AGD_AF_*, mm	16.5	3.9	15.0	6.0	25.0
AGD_AC_**, mm	42.6	6.2	40.0	27.0	60.0
AGD ratio***	0.39	0.08	0.38	0.20	0.57

^*^, **, ***: Student’s t-test; *p* < 0.001

**Table 2:** Percentiles of AGD among males and females

Percentile	Male			Female		
	**AGD**_**AS**_** (mm)**	**AGD**_**AP**_** (mm)**	**AGD ratio**	**AGD**_**AF**_** (mm)**	**AGD**_**AC**_** (mm)**	**AGD ratio**
3	15	40	0.33	10	30	0.22
10	20	45	0.38	10	35	0.25
50	25	50	0.50	15	40	0.38
90	30	60	0.60	20	50	0.50
97	35	70	0.62	25	55	0.50

*Abbreviations*: *AGD*_*AP*_ Anopenile distance, *AGD*_*AS*_ Anoscrotal distance, *AGD*_*AC*_ Anoclitoral distance, *AGD*_*AF*_ Anofourchette distance

AGD ratio: AGD_AS_/AGD_AP_ (male), AGD_AF_/AGD_AC_ (female)

Table 3 presents the findings of Pearson's correlation analysis used to determine the correlation between AGD parameters and anthropometrics. There was no correlation between the AGD ratio and birth weight or length in both sexes. The AGD_AS_, AGD_AP,_ AGD_AF_, and AGD_AC_, however, had a statistically significant but weak positive correlation with birth weight and length.

**Table 3:** Correlation of AGD and AGD ratio with anthropometric parameters

	**BW (Kg)**		**Length (cm)**		**HC (cm)**	
	**r**	***p* value**	**r**	***p* value**	**r**	***p* value**
**Male**						
AGD_AS_ (mm)	0.265	** < 0.001**	0.259	** < 0.001**	0.032	0.665
AGD_AP_ (mm)	0.235	** < 0.001**	0.260	** < 0.001**	0.076	0.299
AGD ratio	0.104	0.153	0.077	0.295	-0.039	0.592
**Female**						
AGD_AF_ (mm)	0.433	** < 0.001**	0.311	** < 0.001**	0.146	0.05
AGD_AC_ (mm)	0.340	** < 0.001**	0.249	**0.001**	0.232	**0.002**
AGD ratio	0.081	0.276	0.070	0.352	0.166	**0.025**

Pearson’s correlation coefficient (r) was used to assess the correlation

*Abbreviations*: *AGD*_*AP*_ Anopenile distance, *AGD*_*AS*_ Anoscrotal distance, *AGD*_*AC*_ Anoclitoral distance, *AGD*_*AF*_ Anofourchette distance

AGD ratio: AGD_AS_/AGD_AP_ (male), AGD_AF_/AGD_AC_ (female)

## Discussion

AGD is a marker to assess under-androgenization or over-androgenization. Male infants tend to have a shorter AGD than predicted if they have undescended testes, hypospadias, or prenatal exposure to antiandrogenic endocrine disrupting chemicals such as phthalate [9, 10]. In contrast, longer AGD can be found in virilized females. Female babies with virilizing congenital adrenal hyperplasia were found to have labioscrotal fusion and an increased anogenital ratio [3]. A study demonstrated that maternal cigarette smoking is associated with increased weight-adjusted AGD in female neonates [11]. This is likely due to the suppression of placental aromatase, a key enzyme responsible for the conversion of androgen to estrogen [12, 13].

The present study showed that there was a significant difference between AGD parameters in males and females. Sexual dimorphism of AGD is observed as males having a longer AGD than females. These findings may be explained by the fact that external genitalia are developed under the influence of sex hormones [14], particularly during the masculinization programming window which occurs between 8 and 14 weeks of pregnancy in humans [15]. Because of this period of prenatal androgen action, the distance between the anus and the base of the genital tubercle is approximately twice as long in males as females in both rodents and humans [16]; however, there are some variations, as Özkan B. et al. [17] found that the ratio of AGD_AS_ in males to AGD_AF_ in females was 2.2, while it is around 1.6 in our study.

The AGD measurements in term newborns from different countries are presented in Table 4. The mean AGD_AS_ in male (25.2 mm.) and AGD_AF_ in female (16.5 mm.) in the present study are comparable with the data from the USA reported by Sathyanarayana S and colleagues (mean AGD_AS_ 24.7 mm. and mean AGD_AF_ 16.0 mm.). [8], but much higher than the mean value reported by Shah R. et al. [18] (mean AGD_AS_ 21.0 mm. and mean AGD_AF_ 13.0 mm.) and the UK study [16] (mean AGD_AS_ 19.8 mm. and mean AGD_AF_ 9.1 mm.). The variation in AGD parameters between studies may be explained by ethnic differences, equipment, systematic errors in measurement, and measurement protocols.

**Table 4:** Literature review of AGD reference ranges in term newborns of both sexes among different countries

Countries	Female			Male		
	AGD_AF_ (mm)	AGD_AC_ (mm)	AGD ratio	AGD_AS_ (mm)	AGD_AP_ (mm)	AGD ratio
USA [18] (2021)	13.0 ± 2.0	35.0 ± 3.0	0.37 ± 0.07	21.0 ± 4.0	50.0 ± 4.0	0.42 ± 0.07
USA [8] (2015)	16.0 ± 3.2	36.7 ± 3.9	-	24.7 ± 4.5	49.6 ± 5.9	-
European countries (Multicenter) [19] (2020)	14.8 ± 3.5	37.8 ± 4.5	0.39 ± 0.10	24.6 ± 4.7	47.6 ± 5.8	0.49 ± 0.10
Turkey [17] (2011)	10.3 ± 0.2	30.0 ± 0.2	0.30 ± 0.10	23.0 ± 0.6	56.0 ± 1.0	0.48 ± 0.80
Ghana [5] (2017)	13.6 ± 2.7	34.2 ± 3.3	-	25.5 ± 5.0	48.9 ± 5.6	-
UK [16] (2009)	9.1 ± 2.8	-	-	19.8 ± 6.1	-	-
Nigeria [20] (2019)	-	-	-	25.5 ± 3.9	48.7 ± 3.9	-
Korea [21] (2015)	-	-	-	23.0 ± 2.0	42.0 ± 3.0	-
Present study	16.5 ± 3.9	42.6 ± 6.2	0.39 ± 0.08	25.2 ± 4.8	52.6 ± 6.9	0.48 ± 0.08
**Summary**^a^	**13.2 ± 2.9**	**35.9 ± 3.9**	**0.37 ± 0.09**	**23.7 ± 4.8**	**49.7 ± 5.2**	**0.47 ± 0.36**

Values are expressed as mean ± SD

^a^Weighted mean ± pooled variance

*Abbreviations*: *AGD*_*AP*_ Anopenile distance, *AGD*_*AS*_ Anoscrotal distance, *AGD*_*AC*_ Anoclitoral distance, *AGD*_*AF*_ Anofourchette distance

AGD ratio: AGD_AS_/AGD_AP_ (male), AGD_AF_/AGD_AC_ (female)

Various infant positionings were used during AGD measurement in previous studies, which may result in different AGD values. The two most common technique from the literature are the TIDES method and the Cambridge method. Both protocols require the assistance of another person to hold the infant in a supine position, but the differences are in the posture of the lower half of the body. The TIDES method places the newborn's legs in a frog-like position and pulls the knees back toward the shoulders, whereas the Cambridge method places both hips in flexion, puts the feet on the surface, and exerts light pressure onto the thighs [22]. The TIDES method creates a slight stretch of the newborn's perineum during measurement, resulting in a longer AGD value than the Cambridge method [22]. In this study, AGD parameters were assessed using only a position similar to the TIDES technique; therefore, future research may be needed to develop the position-specific reference ranges for AGD.

Our findings support previous study that found significant but weak positive correlations between AGD_AS_, AGD_AP_, AGD_AF_, AGD_AC_, and birth weight and length in term newborns [5]. In that study, the strongest correlation was found between AGD_AS_ and birth weight in male newborns (*r* = 0.306; *p* < 0.001), whereas our study found the strongest correlation between AGD_AF_ and birth weight in female newborns (*r* = 0.433; *p* < 0.001). Mondal, et al. [23] measured the AGD_AF_ in term and preterm female newborns in India and found a weak positive correlation between the AGD_AF_ and birth weight (*r* = 0.232, *p* < 0.001), length (*r* = 0.165, *p* = 0.008), and head circumference (*r* = 0.225, *p* < 0.001). A number of studies have also discovered variable positive correlations between AGD and birth weight and length [3, 16, 19, 20].

Despite the fact that the mean AGD_AS_, AGD_AP_, AGD_AF_, and AGD_AC_ varied across studies, we found a consistent AGD ratio [8, 16–21]. Ranges of mean AGD ratio in female and male newborns were 0.30–0.39 and 0.42–0.49, respectively. In our study, the mean (± SD) AGD ratio is 0.48 ± 0.08 and 0.39 ± 0.08 in males and females, closest to what was reported in a European multicenter study of 686 term babies which also found a significant difference between AGD ratios in males (0.49 ± 0.1) and females (0.39 ± 0.1) and intermediate values in differences of sex development (DSD) (0.43 ± 0.1) [19]. Moreover, AGD ratio, unlike other AGD parameters, was unaffected by birth weight or length, suggesting that it might be a better marker for determining the degree of prenatal androgen exposure in a full-term newborn than the distance measures alone. However, the AGD ratios in Table 4 were largely from southeastern Europe and southwestern Asia, and the availability of the data in full-term newborns from other regions are limited, which could impact the generalizability of the AGD ratio values, therefore, more study on the AGD ratio is necessary.

AGD can be utilized to gain insight into the effect of androgens during pregnancy, to assess newborns with DSD, and to assess the health implications of endocrine disruptors in environmental toxicology. Normative data on AGD ranges for local references should be established as standards for comparison in clinical practice. We offered five percentile thresholds in our results as an initial standard for Thailand.

This study is the first to present data and standards for term newborns in Thailand. The limitation of our research is the single-center study and the reliance on a single measurement of AGD. However, the reliability of this measurement in humans has been well-established, and since one examiner performed all of the measurements, interobserver errors were minimized.

## Conclusion

The present study provided sex-specific ranges and ratio for AGD in Thai healthy full-term newborns. We proposed using the AGD ratio, instead of individual AGD, as an indicator of prenatal androgen exposure.

## Data Availability

The datasets generated and/or analyzed during the current study are not publicly available due ethical issue but are available from the corresponding author on reasonable request.

## Abbreviations

AGD_AP_: Anopenile distance
AGD_AS_: Anoscrotal distance
AGD_AC_: Anoclitoral distance
AGD_AF_: Anofourchette distance
AGD ratio: AGD_AS_/AGD_AP_ (male), AGD_AF_/AGD_AC_ (female)
